# Acute cold hypersensitivity characteristically induced by oxaliplatin is caused by the enhanced responsiveness of TRPA1 in mice

**DOI:** 10.1186/1744-8069-8-55

**Published:** 2012-07-28

**Authors:** Meng Zhao, Kouichi Isami, Saki Nakamura, Hisashi Shirakawa, Takayuki Nakagawa, Shuji Kaneko

**Affiliations:** 1 Department of Molecular Pharmacology, Graduate School of Pharmaceutical Sciences, Kyoto University, 46-29 Yoshida-Shimoadachi-cho, Sakyo-ku, Kyoto 606-8501, Japan

**Keywords:** Chemotherapy, Numbness, Pain, Peripheral neuropathy, Sensitization, TRP channels

## Abstract

**Background:**

Oxaliplatin, a platinum-based chemotherapeutic agent, causes an unusual acute peripheral neuropathy. Oxaliplatin-induced acute peripheral neuropathy appears in almost all patients rapidly after infusion, and is triggered or exacerbated by cold, while its mechanisms are poorly understood. In this study, the involvement of thermosensitive transient receptor potential channels (TRPA1, TRPM8 and TRPV1) in oxaliplatin-induced acute hypersensitivity was investigated in mice.

**Results:**

A single intraperitoneal administration of oxaliplatin (1–10 mg/kg) induced cold but not mechanical hypersensitivity within 2 h in a dose-dependent manner. Infusion of the oxaliplatin metabolite, oxalate (1.7 mg/kg), also induced acute cold hypersensitivity, while another platinum-based chemotherapeutic agent, cisplatin (5 mg/kg), or the non-platinum-containing chemotherapeutic agent, paclitaxel (6 mg/kg) failed to induce mechanical or cold hypersensitivity. The oxaliplatin-induced acute cold hypersensitivity was abolished by the TRPA1 antagonist HC-030031 (100 mg/kg) and by TRPA1 deficiency. The nocifensive behaviors evoked by intraplantar injections of allyl-isothiocyanate (AITC; TRPA1 agonist) were significantly enhanced in mice treated for 2 h with oxaliplatin (1–10 mg/kg) in a dose-dependent manner, while capsaicin (TRPV1 agonist)-evoked nocifensive behaviors were not affected. Menthol (TRPM8/TRPA1 agonist)-evoked nocifensive-like behaviors were also enhanced by oxaliplatin pretreatment, which were inhibited by TRPA1 deficiency. Similarly, oxalate enhanced, but neither cisplatin nor paclitaxel affected AITC-evoked nocifensive behaviors. Pretreatment of cultured mouse dorsal root ganglia (DRG) neurons with oxaliplatin (30–300 μM) for 1, 2, or 4 h significantly increased the number of AITC-sensitive neurons in a concentration-dependent manner whereas there was no change in the number of menthol- or capsaicin-sensitive neurons.

**Conclusions:**

Taken together, these results suggest that a brief treatment with oxaliplatin or its metabolite oxalate is sufficient to enhance the responsiveness of TRPA1 but not that of TRPM8 and TRPV1 expressed by DRG neurons, which may contribute to the characteristic acute peripheral neuropathy induced by oxaliplatin.

## Background

Oxaliplatin, a third-generation, platinum-based chemotherapeutic agent, has superior activity as a first-line treatment in advanced colorectal cancer [1] and as adjuvant treatment [2]. Oxaliplatin has a better safety profile, characterized by lower hematotoxicity and manageable gastrointestinal toxicity, than other platinum-based chemotherapeutics. However, peripheral neuropathy is a common side effect of platinum-based chemotherapeutic compounds such as oxaliplatin and cisplatin, taxanes such as paclitaxel and vinca alkaloids such as vincristine [3]. Oxaliplatin induces moderate to severe peripheral neuropathy, characterized by two types of neurological symptoms [4,5]. During or within hours after its infusion, an acute neuropathy, including acral numbness, paresthesia, dysesthesia and pain, develops in almost all patients that intensifies over time, causing serious discomfort. The acute neuropathy is specific to oxaliplatin and is triggered or exacerbated by cold [4-6]. After multiple chemotherapy cycles, chronic cumulative peripheral neuropathy, such as sensory loss and motor dysfunction, occurs in 10–15% of treated patients, a rate similar to that of cisplatin [5,7]. While the oxaliplatin-induced chronic peripheral neuropathy can be explained, at least in part, by the accumulation of platinum adducts in the dorsal root ganglia (DRG) [8], the mechanisms underlying the acute peripheral neuropathy are poorly understood.

Sensory neurons express several types of transient receptor potential (TRP) channels, including TRPV1, TRPA1, and TRPM8. These receptors are thermosensitive and play a critical role in pain generation [9,10]. TRPV1 is activated by noxious heat, acidity, and noxious chemical stimuli such as capsaicin [11,12]. TRPA1 is activated by noxious cold and a large number of irritants including allyl-isothiocyanate (AITC), cinnamaldehyde, allicin, and aldehydes, as well as reactive-oxygen and nitrogen species [13-16]. TRPM8 is activated by innocuous as well as noxious cold, and by menthol, the ingredient of peppermint that produces its cooling sensation [17,18]. Although still a matter of debate, recent evidence suggests that these thermosensitive TRP channels are responsible for chemotherapy-induced peripheral neuropathies. In rodents, oxaliplatin increases the expression of TRPA1 [19-21] and TRPM8 [22], but see [19,21], but not TRPV1 [21], mRNAs in sensory ganglia. In mouse trigeminal ganglia, cisplatin increases TRPV1 and TRPA1 mRNAs levels [21], but see [23]. Oxaliplatin-induced mechanical and cold hypersensitivity is abolished by pharmacological inhibition or a gene-deficiency of TRPA1 [19,20], TRPV1 [21], or TRPM8 [19,22], but see [23], and cisplatin- and paclitaxel-induced painful neuropathy by an antagonist or genetic deficiency of TRPV1 [21,24,25]. In those studies, chemotherapy-induced hypersensitivity was assessed several days to several weeks after a single or repeated administration of the compound, which may reflect the subacute or chronic phase of chemotherapy-induced peripheral neuropathy.

In this study, we used a mouse model to investigate whether TRPA1, TRPM8, and TRPV1 are involved in the oxaliplatin-induced acute peripheral neuropathy. Here, we show that, within hours, a single administration of oxaliplatin, but not of cisplatin or paclitaxel, induces a cold hypersensitivity that is associated with an enhanced responsiveness of TRPA1, but not of TRPM8 and TRPV1, on DRG neurons.

## Results

Effect of oxaliplatin, cisplatin, and paclitaxel on acute mechanical and cold sensitivities.

**Figure 1:**
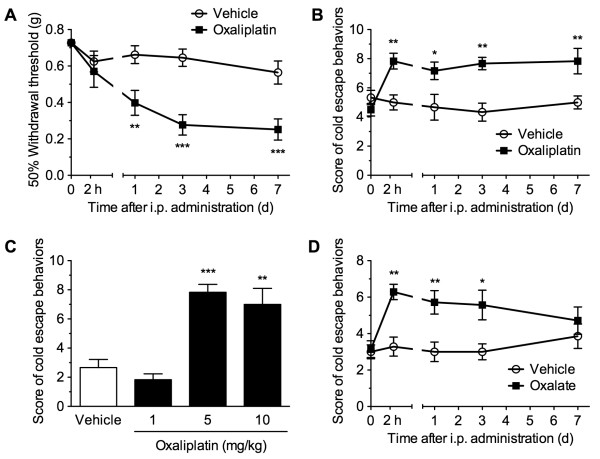
**A single administration of oxaliplatin induces acute cold hypersensitivity, but not acute mechanical hypersensitivity.** ( **A**, **B**) Mice were intraperitoneally administered either vehicle or oxaliplatin (5 mg/kg). At the indicated times, the 50% withdrawal threshold to mechanical stimulation and escape behaviors in response to cold stimulation (5°C) were evaluated in a von Frey filament test ( **A**, *n* = 8) and a cold-plate test ( **B**, *n* = 6), respectively. Cold escape behaviors were scored depending on a behavioral assessment, with the total score calculated for a period of 60 s. * *p* < 0.05, ** *p* < 0.01, *** *p* < 0.001 compared with vehicle-administered group. ( **C**) Mice were intraperitoneally administered vehicle or oxaliplatin (1, 5 or 10 mg/kg). Two hours after the administration, the cold escape behaviors were scored in the cold-plate test. *n* = 6. ** *p* < 0.01; *** *p* < 0.001 compared with vehicle-administered group. ( **D**) Mice were intraperitoneally administered either vehicle or oxalate (1.7 mg/kg). At the indicated times, the cold escape behaviors were scored in the cold-plate test. *n* = 7. * *p* < 0.05 compared with vehicle-administered group. Data are presented as the means ± S.E.M. Statistical significance was calculated by one-way ANOVA (C) or two-way repeated measures ANOVA, followed by Bonferroni post-hoc test.

The effects of a single administration of oxaliplatin on behavioral sensitivity to mechanical and cold stimuli were assessed in a von Frey filament test and a cold-plate test, respectively (Figure 1). A single intraperitoneal administration of oxaliplatin (5 mg/kg) significantly decreased the 50% withdrawal threshold to mechanical stimulation with von Frey filaments (*F*_1,14_ = 16.85, *p* < 0.01). While a decrease in the mechanical threshold was not apparent 2 h after oxaliplatin administration, the response became significant at 1 day and lasted for at least 7 days, compared with the vehicle-administered group (Figure 1A). In the cold-plate test, oxaliplatin (5 mg/kg) significantly increased the escape behavior scores measured in response to cold stimulation (*F*_1,10_ = 10.06, *p* < 0.01). Significant increases in the cold escape behaviors were observed after 2 h and lasted for at least 7 days after oxaliplatin administration, compared with the vehicle-administered group (Figure 1B). We assessed the dose-dependent effect of oxaliplatin on the acute cold hypersensitivity in the cold-plate test. The cold escape behavior scores were significantly increased 2 h after the administration of oxaliplatin (1, 5 and 10 mg/kg) in a dose-dependent manner (*F*_3,20_ = 18.57, *p* < 0.001), and the significant effects were observed at doses of 5 and 10 mg/kg, but not 1 mg/kg, compared with the vehicle-administered group (Figure 1C). Oxaliplatin is metabolized to oxalate and dichloro(1,2-diaminocyclohexane)platinum. As oxaliplatin-induced cold hypersensitivity measured in the acetone test is caused, at least in part, by oxalate [26], we examined the effect of oxalate on the cold sensitivity in the cold-plate test. The dose of sodium oxalate (1.7 mg/kg) was calculated from the molecular weight of oxaliplatin included in the oxaliplatin preparation (5 mg/kg). A single intraperitoneal administration of oxalate significantly increased the cold escape behavior scores (*F*_1,12_ = 14.81, *p* < 0.01). Significant increases were observed after 2 h and lasted for 3 days after oxaliplatin administration, compared with the vehicle-administered group (Figure 1D).

**Figure 2:**
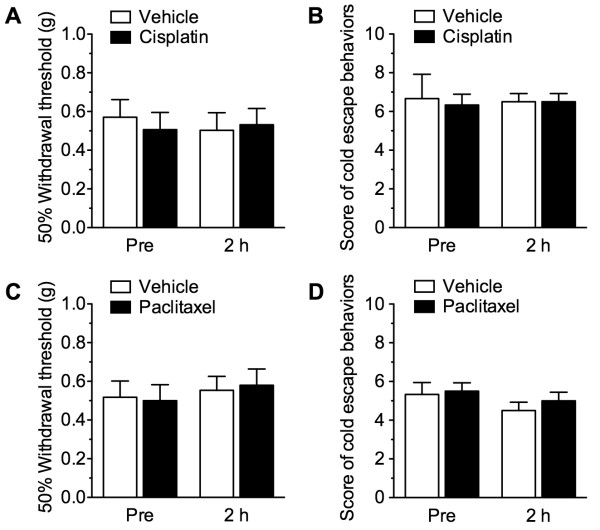
**Neither cisplatin nor paclitaxel induces acute mechanical and cold hypersensitivity.** Mice were intraperitoneally administered vehicle, cisplatin (5 mg/kg; **A**, **B**), or paclitaxel (6 mg/kg; **C**, **D**), and 2 h later the 50% withdrawal threshold to mechanical stimulation ( **A**, **C**) and the escape behaviors in response to cold stimulation ( **B**, **D**) were evaluated. Data are presented as the means ± S.E.M. of 6 mice.

The repeated administration of cisplatin or paclitaxel is reported to produce mechanical and thermal hypersensitivity, with maximal effects observed several days to several weeks after drug administration [24,25,27,28]. In this study, we asked whether cisplatin and paclitaxel induce an early-phase acute mechanical or cold hypersensitivity. However, 2 h after a single intraperitoneal administration of cisplatin (5 mg/kg) or paclitaxel (6 mg/kg) there was no change in the 50% withdrawal threshold to mechanical stimulation) or in the escape behavior scores measured in response to cold stimulation (Figures 2).

Involvement of TRPA1 in acute oxaliplatin-induced cold hypersensitivity.

**Figure 3:**
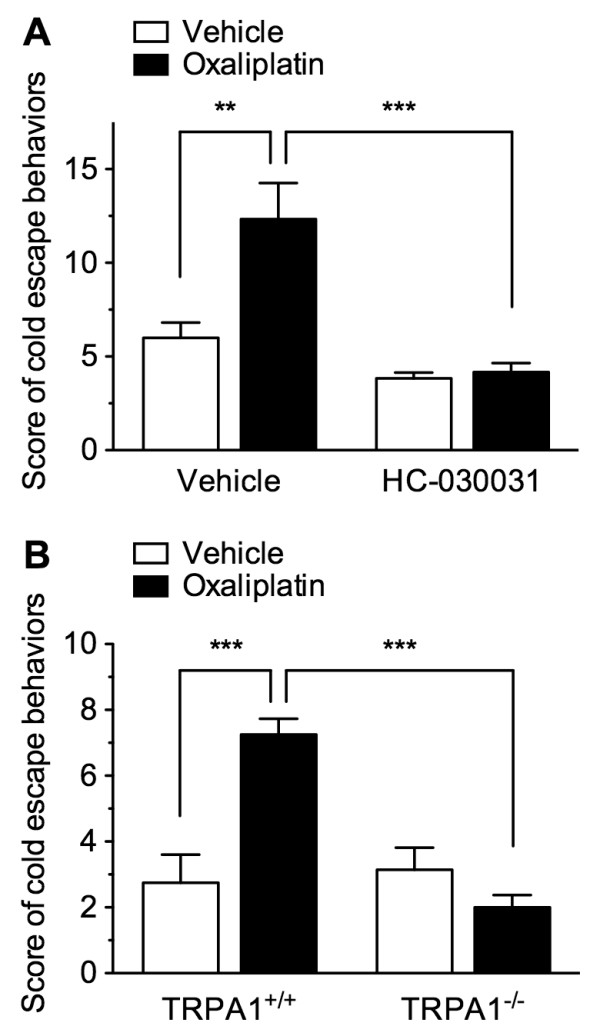
**TRPA1 is involved in oxaliplatin-induced acute cold hypersensitivity.** ( **A**) Mice were intraperitoneally administered vehicle or oxaliplatin (5 mg/kg) 2 h before the cold-plate test, with vehicle or HC-030031 (100 mg/kg) intraperitoneally injected 30 min before testing. *n* = 6. ( **B**) TRPA1^+/+^ or TRPA1^−/−^ mice were intraperitoneally administered vehicle or oxaliplatin (5 mg/kg) 2 h before the cold-plate test. *n* = 7–8. Escape behaviors in response to cold stimulation (5°C) were scored in a cold-plate test. Data are presented as the mean ± S.E.M. ** *p* < 0.01, *** *p* < 0.001.

To determine whether TRPA1 is involved in acute oxaliplatin-induced cold hypersensitivity, we examined the effects of a TRPA1 antagonist and of TRPA1 deficiency. In the cold-plate test, escape behavior scores measured in response to cold stimulation were significantly higher in mice tested 2 h after oxaliplatin (5 mg/kg, i.p.) administration than in mice tested 2 h after vehicle administration. In 2 h vehicle-administered mice, an i.p. injection of the TRPA1 antagonist HC-030031 (100 mg/kg) 30 min before the cold-plate test tended to decrease the cold escape behavior scores, although the effect was not significant. In 2-h oxaliplatin-administered mice, HC-030031 significantly inhibited oxaliplatin-induced acute cold hypersensitivity compared to 30-min vehicle-injected mice (Figure 3A). Similarly, *Trpa1*^+/+^ mice exhibited acute cold hypersensitivity 2 h after oxaliplatin (5 mg/kg) administration, whereas the response was completely abolished in *Trpa1*^−/−^ mice (Figure 3B).

Effects of oxaliplatin, cisplatin, and paclitaxel on AITC-, menthol- and capsaicin-evoked nocifensive behaviors.

**Figure 4:**
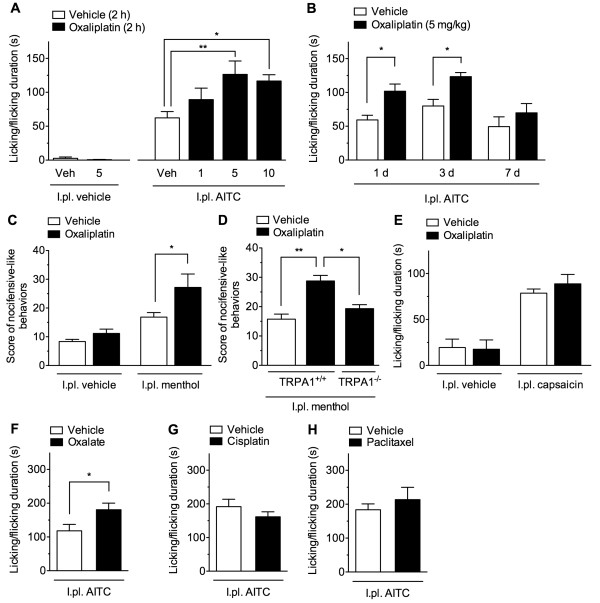
**A single administration of oxaliplatin, but not cisplatin and paclitaxel, enhances AITC-evoked nocifensive behaviors.** ( **A**) Two hours after a single administration of vehicle or oxaliplatin (1, 5 or 10 mg/kg), mice received an intraplantar injection of vehicle ( *n* = 3) or AITC (0.1%, 20 μl; *n* = 7–14). ( **B**) 1, 3 or 7 days after a single administration of vehicle or oxaliplatin (5 mg/kg), mice received an intraplantar injection of AITC (0.1%, 20 μl). *n* = 5–6. ( **C**) Two hours after a single administration of vehicle or oxaliplatin (5 mg/kg), mice received an intraplantar injection of vehicle or menthol (1.6 μg, 20 μl). *n* = 6. ( **D**) TRPA1^+/+^ or TRPA1^−/−^ mice were intraperitoneally administered vehicle or oxaliplatin (5 mg/kg) 2 h before the menthol-evoked nocifensive behaviors. *n* = 3–4. ( **E**) Two hours after a single administration of vehicle or oxaliplatin (5 mg/kg), mice received an intraplantar injection of vehicle or capsaicin (160 μg, 20 μl). *n* = 6. ( **F**– **G**) Two hours after a single administration of oxalate (1.7 mg/kg; **F**), cisplatin (5 mg/kg; **G**), paclitaxel (6 mg/kg; **H**) or vehicle, mice received an intraplantar injection of AITC (0.1%, 20 μl). *n* = 6–7. AITC- and capsaicin-evoked nocifensive behaviors were measured for 20 min and 5 min, respectively; menthol-evoked nocifensive-like behaviors were scored for 5 min. Data are presented as the means ± S.E.M. * *p* < 0.05; ** *p* < 0.01; *** *p* < 0.001.

To investigate the involvement of thermosensitive TRP channels, we assessed the effects of oxaliplatin, cisplatin, and paclitaxel on the nocifensive behaviors evoked by TRP channel agonists. Intraplantar (i.pl.) injection of AITC (0.1%, 20 μl), a TRPA1 agonist, evoked nocifensive behaviors such as licking and flicking of the injected hindpaw, whereas this was not the case following i.pl. vehicle injection. When mice were preinjected with oxaliplatin (1, 5 and 10 mg/kg, i.p.) and then tested 2 h later, the duration of the AITC-evoked nocifensive behaviors were significantly enhanced in a dose-dependent manner (*F*_3,31_ = 5.711, *p* < 0.001). The significant differences were observed at doses of 5 and 10 mg/kg, but not 1 mg/kg, compared with mice preinjected with vehicle (Figure 4A). We examined the time course of the oxaliplatin-induced enhancement of the AITC-evoked nocifensive behaviors. The duration of the AITC-evoked nocifensive behaviors were significantly enhanced even 1 and 3 days, but not 7 days after a single administration of oxaliplatin (5 mg/kg, i.p.), compared with mice preinjected with vehicle (Figure 4B).

Intraplantar injection of menthol (160 μg), a TRPM8/TRPA1 agonist [29], evoked nocifensive-like behaviors, such as backwards walking and lifting of the injected hindpaw. In mice preinjected with oxaliplatin 2 h prior to testing, the scores of menthol-evoked nocifensive-like behaviors were significantly higher than those of vehicle-administered mice (Figure 4C). The enhancement of menthol-evoked nocifensive-like behaviors induced by oxaliplatin pretreatment was significantly inhibited in *Trpa1*^*−/−*^ mice to the scores in vehicle-pretreated mice (Figure 4D). Intraplantar injection of the TRPV1 agonist capsaicin (1.6 μg) evoked nocifensive behaviors such as licking and flicking of the injected hindpaw. There was no significant difference in the duration of the capsaicin-evoked nocifensive behaviors between vehicle- and oxaliplatin-preinjected mice (Figure 4E).

When mice were preinjected with oxalate (1.7 mg/kg, i.p.) and then tested 2 h later, the duration of the AITC-evoked nocifensive behaviors was significantly enhanced compared with mice preinjected with vehicle (Figure 4F). By contrast, a 2-h pre-injection of cisplatin (5 mg/kg, i.p.) or paclitaxel (6 mg/kg, i.p.) had no effects on the duration of the AITC-evoked nocifensive behaviors (Figures 4G, H).

Oxaliplatin enhances the response to TRPA1 agonist, but not that of TRPM8 and TRPV1 agonists, in cultured DRG neurons.

**Figure 5:**
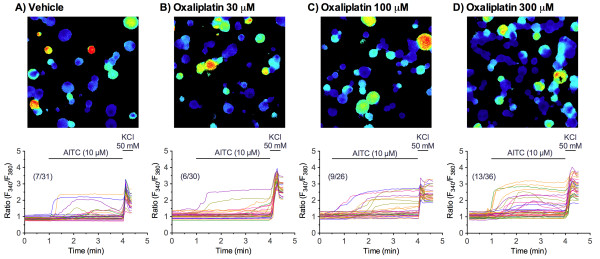
**Oxaliplatin pretreatment increases the AITC-evoked Ca**^**2+**^**response in cultured DRG neurons.** The cultured neurons were pretreated with vehicle ( **A**) or oxaliplatin at concentrations of 30 ( **B**), 100 ( **C**) or 300 μM ( **D**) for 2 h after which AITC (10 μM) was added for 3 min. Representative image of fluorescence (upper panels) and Ca^2+^ concentration (F_340_/F_380_ ratio) recorded in individual cultured DRG neuron (lower panels). All neurons in the fields were identified based on the [Ca^2+^]_i_ increase elicited by the application of 50 mM KCl. Cell numbers in (AITC-sensitive DRG neurons)/(Total counted neurons) in the representative data are indicated in parentheses in lower panels.

**Figure 6:**
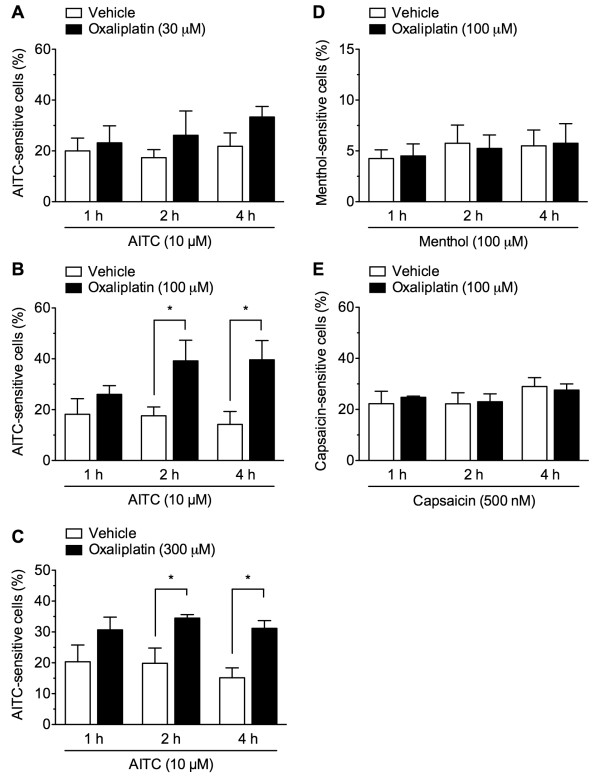
**Oxaliplatin pretreatment increases the number of AITC-sensitive DRG neurons.** ( **A**- **C**) In cultured DRG neurons pretreated with vehicle or oxaliplatin at concentrations of 30 ( **A**), 100 ( **B**) or 300 μM ( **C**) for 1, 2, or 4 h, AITC (10 μM) was added for 3 min. ( **D**, **E**) In cultured DRG neurons pretreated with vehicle or oxaliplatin (100 μM) for 1, 2, or 4 h, menthol (100 μM; **D**) or capsaicin (500 nM; **E**) was added for 3 min, and the Ca^2+^ responses of the neurons were determined. Cells were considered responsive if their F_340_/F_380_ ratio increased by more than 0.2 during the 3-min application. The values show the percentage of agonist-sensitive cells in 50 mM KCl-positive neurons. Data are presented as the mean ± S.E.M. of 4–6 separate experiments. *p < 0.05, compared with corresponding vehicle-pretreatment.

The effects of oxaliplatin pretreatment on the responses to TRPA1, TRPM8, and TRPV1 agonists in cultured DRG neurons isolated from naïve mice were assessed by calcium imaging. An application of the TRPA1 agonist AITC at concentrations of 1, 10 and 100 μM to naïve cultured DRG neurons concentration-dependently evoked a [Ca^2+^_i_ increase in approximately 5.0 ± 1.3%, 22.2 ± 3.2% and 38.2 ± 2.8% of the cells, respectively, consistent with previous reports (25–45%) [29-31]. To better detect alterations of TRPA1 function, cultured DRGs were treated with a relatively low AITC concentration of 10 μM. In cultured DRG neurons pretreated with oxaliplatin (30, 100 and 300 μM) for 2 h, the numbers of 10 μM AITC-sensitive cells were increased in a concentration-dependent manner, although the amplitudes of increase in F_340_/F_380_ ratio seemed to be less or not increased (Figure 5). We quantitatively assessed the numbers of AITC-sensitive cells. In cultured DRG neurons pretreated with oxaliplatin (30 μM) for 1, 2 and 4 h, the numbers of AITC-sensitive cells were not changed, compared with those pretreated with vehicle for 1, 2 and 4 h (*F*_1,30_ = 2.55, *P* = 0.121) (Figure 6A). By contrast, the numbers of AITC-sensitive cells pretreated with oxaliplatin (100 or 300 μM) for 1, 2 and 4 h were significantly increased (*F*_1,24_ = 14,24, *P* < 0.001 and *F*_1,30_ = 18.85, *P* < 0.001, respectively). Although the changes became apparent at 1 h, significant differences reached at 2 and 4 h of oxaliplatin pretreatment (Figure 6B, C).

An application of menthol (100 μM) evoked a [Ca^2+^_i_ increase in 4–6% of cultured DRG neurons pretreated with vehicle, which was consistent with (4.2-7% [32-34]) or less than previous reports (10-17% [17,23,30,35,36]). In cultured DRG neurons pretreated with oxaliplatin (100 μM) for 1, 2, or 4 h, there was no change in the number of menthol-sensitive cells at any time point (Figure 6D). Similarly, an application of capsaicin at a relatively low concentration of 500 nM evoked a [Ca^2+^_i_ increase in 22–29% of vehicle-treated cultured DRG neurons, with no change in the number of capsaicin-sensitive cells at any time point in those pretreated with oxaliplatin (Figure 6E).

## Discussion

In the present study, we provide the first evidence that TRPA1 in DRG neurons mediates the acute phase of oxaliplatin-induced peripheral neuropathy, as supported by the following results. 1) A single administration of oxaliplatin, as well as its metabolite oxalate, produced rapid-onset cold hypersensitivity within 2 h; this response was blocked by a TRPA1 antagonist and by TRPA1 deficiency. 2) Nocifensive behaviors evoked by AITC and menthol, but not by capsaicin, were enhanced 2 h after oxaliplatin administration. 3) Pretreatment of the cultured DRG neurons with oxaliplatin for 2–4 h increased the number of AITC-, but not of menthol- and capsaicin-sensitive neurons.

The peripheral neuropathy caused by chemotherapeutic agents, including oxaliplatin, has been widely evaluated experimentally in rodents as hypersensitivity to mechanical and thermal stimuli in terms of mechanical allodynia and thermal hyperalgesia, respectively. Previous studies in animal models largely focus on the oxaliplatin-induced chronic painful neuropathy that appears several days to several weeks after oxaliplatin administration [19-22,37], while oxaliplatin-induced acute neuropathy is less well characterized [26]. Our findings in mice, in which cold hypersensitivity was detected as early as 2 h after oxaliplatin administration, is consistent with the clinical observation of a characteristic acute sensory neuropathy triggered by cold that appears during or within hours of oxaliplatin infusion. By contrast, mechanical hypersensitivity in mice was observed 1 day, but not as early as 2 h, after drug administration and persisted for at least 7 days, consistent with previous reports [20,26,37]. Moreover, the rapid-onset cold hypersensitivity was not produced by another platinum-based chemotherapeutic agent, cisplatin, or by the non-platinum-containing chemotherapeutic agent, paclitaxel, both of which are known to induce chronic peripheral neuropathy following repeated administration [24,25,27,28]. These findings suggest that the rapid-onset cold hypersensitivity is representative of the acute peripheral sensory neuropathy characteristic to oxaliplatin in mice and that the mouse model is suitable for evaluating the mechanisms of this side effect.

The major finding of this study is that oxaliplatin leads to the selective enhancement of TRPA1-mediated responses within a relatively short time, both in vivo and in vitro. AITC-evoked nocifensive behaviors and Ca^2+^ influx in DRG neurons are mediated through the activation of TRPA1 [29,38]. Oxaliplatin increased both of these TRPA1-mediated responses within several hours, suggesting that it rapidly leads to an enhanced TRPA1 responsiveness in sensory neurons. The finding that oxalate enhanced AITC-evoked nocifensive behaviors suggests that the rapid-onset effects characteristic of oxaliplatin are caused by its metabolite, oxalate, or by an oxalate-related structure of oxaliplatin. Consistent with our findings, Sakurai et al. showed that both oxaliplatin and oxalate induce an early-phase (several hours) cold hyperalgesia in the acetone test, whereas a late-phase mechanical allodynia is induced by oxaliplatin or its another metabolite dichloro(1,2-diaminocyclohexane)platinum, but not oxalate, in rats [26]. On the other hand, the oxaliplatin-induced enhancement of AITC-evoked nocifensive behaviors lasted, at least, 3 days after the administration, suggesting that the enhanced TRPA1 responsiveness contributes to not only the acute (several hours), but also, at least, subacute (several days) oxaliplatin-induced cold hypersensitivity.

Despite some controversies, recent evidence points to the involvement of TRPA1 in oxaliplatin-induced subacute and chronic peripheral neuropathy [19-21,39]. Subacute (several days) mechanical and cold hypersensitivities induced by a single administration of oxaliplatin were shown to be inhibited by either a TRPA1 antagonist or TRPA1 deficiency [19,20], while the antagonist failed to inhibit the oxaliplatin-enhanced cold-temperature avoidance behavior [19]. Furthermore, subacute (several days) and chronic (several weeks) administration of oxaliplatin increases TRPA1 mRNA levels in DRGs and in the trigeminal ganglion [19,21]. Controversially, a transient up-regulation of TRPA1 mRNA is observed in DRGs only 6 h after a single administration of oxaliplatin [20]. However, it is unlikely that oxaliplatin is able to increase the expression of functional TRPA1 protein within several hours of its administration. In the Ca^2+^ imaging experiments of the present study, the AITC concentration was set relatively low (10 μM). Nonetheless, following oxaliplatin treatment, 10 μM AITC produced nearly the same proportion of sensitive neurons (approximately 40%) as obtained with the submaximal concentration of AITC (100 μM) used in the control. Therefore, it is likely that oxaliplatin acutely produces an enhanced TRPA1 responsiveness by increasing the sensitivity to AITC, i.e., through the sensitization of existing TRPA1 and not by an increase in the number of TRPA1-expressing cells. Under inflammatory conditions, TRPA1 sensitization involves its translocation to the plasma membrane via phospholipase C and protein kinase A (PKA) signaling [40,41]. Acute oxaliplatin may likewise induce the TRPA1 sensitization via PKA signaling [39] or through other mechanisms specific to oxaliplatin and oxalate.

TRPM8 is expressed in a subpopulation of small-diameter sensory neurons, which correlates with responses to cooling and menthol [18,42,43]. Menthol sensation is mainly ascribed to TRPM8, while it also activates TRPA1 in a bimodal manner [29]. In Ca^2+^ imaging experiments, menthol-sensitive DRG neurons are largely abolished in TRPM8^−/−^ mice, although a small population of menthol-sensitive neurons remains, probably via TRPA1 activation [32,44]. Nevertheless, we found no change in the number of menthol-sensitive DRG neurons in response to oxaliplatin, suggesting that it has no acute effect on TRPM8-mediated responses. Although menthol-sensitive DRG neurons mediated thorough TRPA1 activation might be increased by acute oxaliplatin, they may be undetectable probably due to too small population or weak activation of TRPA1 by 100 μM menthol in a bimodal phase [29]. Supporting our findings, oxaliplatin enhances Ca^2+^ responses to icilin (TRPA1/TRPM8 agonist), but not WS12 (TRPM8 selective agonist), in rat DRG neurons [39]. By contrast, our present findings showed that menthol-evoked nocifensive-like behaviors were enhanced after acute oxaliplatin administration, which was inhibited by TRPA1 deficiency. Since it is possible that menthol-evoked nocifensive-like behaviors are mediated through TRPA1 activation [45], they may be increased by the enhanced responsiveness of TRPA1 after acute oxaliplatin administration in the dose of menthol used in this study. Several studies examine the involvement of TRPM8 in oxaliplatin-induced peripheral neuropathy. The subacute, but not chronic, effects of oxaliplatin administration were shown to include a transient up-regulation of TRPM8 mRNA in DRGs [19,21,22]. In TRPM8^−/−^ mice, oxaliplatin-enhanced cold avoidance is abolished, while there is no change in oxaliplatin-induced mechanical hypersensitivity [19]. Pharmacological blockade of TRPM8 has no effect on oxaliplatin-induced subacute cold hypersensitivity [23]. Thus, although TRPM8 involvement in oxaliplatin-induced subacute peripheral neuropathy remains to be clarified, our findings seem to rule out an important role for TRPM8 in oxaliplatin-induced acute peripheral neuropathy.

A body of evidence suggests that TRPV1 plays a role in chemotherapy-induced chronic peripheral neuropathy [21,24,25], similar to the neuropathic pain induced by peripheral nerve injury [46]. However, in the present study, neither cisplatin nor paclitaxel altered capsaicin-evoked, TRPV1-mediated nocifensive behaviors. Furthermore, oxaliplatin had no rapid-onset effect on capsaicin-evoked nocifensive behaviors or the number of capsaicin-sensitive DRG neurons, suggesting that oxaliplatin-induced acute peripheral neuropathy is not mediated by TRPV1. Consistent with the present findings, other studies did not find evidence of TRPV1 involvement in oxaliplatin-induced subacute and chronic cold hypersensitivity [20-22].

Accumulating evidence suggests that oxaliplatin, as a platinum-based drug like cisplatin, induces chronic peripheral neuropathy by its direct and indirect neurotoxic effects on peripheral sensory neurons [5,20]. The painful neurotoxicity may secondarily up-regulate and/or sensitize TRPA1, TRPV1, and TRPM8, as occurs in nerve injury-induced neuropathic pain [46-48]. However, our results indicate that oxaliplatin leads to a rapid, preferentially enhanced responsiveness of TRPA1. Since the rapid effect of the drug is unlikely to be due to its neurotoxicity on sensory neurons, a more likely explanation is an alteration in TRPA1 function, either directly or indirectly, within several hours, although the mechanisms remain unclear.

## Conclusions

The present data suggest that the acute cold hypersensitivity characteristically induced by oxaliplatin could be linked to an enhanced responsiveness of TRPA1, but not TRPM8 and TRPV1, on DRG neurons. This pathway may also be involved in other acute symptoms produced in response to oxaliplatin, such as acral numbness, paresthesia, and dysesthesia. Confirmation of these findings would allow the molecular targeting of TRPA1 in the prevention of oxaliplatin-induced acute peripheral neuropathy.

## Methods

### Animals

The male C57BL/6 J mice aged between 6–8 weeks-old were purchased from Japan SLC (Shizuoka, Japan). For experiments investigating the effects of TRPA1 deficiency, wild-type (*Trpa1*^+/+^) and homozygous (*Trpa1*^−/−^) mouse littermates from heterozygous/heterozygous *Trpa1*^+/−^ mice were used (6- to 8-weeks-old). *Trpa1*^−/−^ mice bred from heterozygous mice with a C57BL/6 × 129 S1 background were obtained from Jackson Laboratory (Bar Harbor, ME) and genotyped as previously described [38]. The *Trpa1*^−/−^ mouse line was backcrossed to C57BL/6 J mice for at least 10 generations. All mice were housed under constant ambient temperature (24 ± 1°C) and humidity (55 ± 10%), with alternate light–dark cycles from 8:00 a.m. to 20:00 p.m.. Food and water were freely available. All experiments were conducted in accordance with the Ethical Guidelines of the Kyoto University Animal Experimentation Committee.

### Drugs

Oxaliplatin and sodium oxalate were purchased from Wako Pure Chemical Industries, Ltd. (Osaka, Japan) and freshly dissolved in 5% glucose solution. *cis*-Diammineplatinum(II) dichloride (cisplatin) and paclitaxel were purchased from Sigma-Aldrich, Inc. (St. Louis, MO, USA). Cisplatin was freshly dissolved in sterile saline, and paclitaxel in Cremophor® EL (Sigma-Aldrich) and dehydrated ethanol (1:1) to obtain a stock solution. Prior to its administration, paclitaxel was further diluted with sterile saline. Mice received a single intraperitoneal administration of oxaliplatin (1, 5 or 10 mg/kg), cisplatin (5 mg/kg), paclitaxel (6 mg/kg), or vehicle. The doses of these chemotherapeutic agents were chosen based on previous reports with a single intraperitoneal administration [19,26,28,37,49,50]. HC-030031 (100 mg/kg, Enzo Life Sciences, Exeter, UK) was prepared in 0.5% methylcellulose (Wako).

### Behavioral tests

#### von Frey filament test

Mechanical sensitivity was assessed by the up-down method using calibrated von Frey filaments, as previously described with slight modifications [51,52]. Mice were acclimated on a wire-mesh floor in Plexiglas cubicles (9 cm L × 5 cm W × 5 cm H) for 1 h, after which mechanical sensitivity was evaluated using a set of four calibrated von Frey fibers (0.07, 0.16, 0.4, 1.0 g; Stoelting Co., Wood Dale, IL USA) applied to the plantar surface of the left hindpaw for a few seconds until they bent slightly. A withdrawal reflex of the hindpaw during stimulation or immediately after stimulus removal was considered a positive response. If a positive response was obtained to the first stimulus, the 0.16-g filament, then the next lower filament was applied; if there was no response, the next higher filament was used. After the first change in responses, the experiment was continued until four additional responses were obtained. The 50% paw withdrawal threshold value was then calculated (Chaplan et al., 1994).

### Cold-plate test

Cold sensitivity was assessed with the hot/cold-plate analgesimeter (Ugo Basile, Milan, Italy). Mice were allowed to acclimate to the testing apparatus for 1 h, after which they were individually placed on the center of a cold plate maintained at 5°C in a transparent Plexiglas cylinder. Escape behaviors were observed for 60 s and graded with a score of 0 = no response; 1 = moderate effort to avoid cold, such as lifting a hindpaw or walking backwards; and 2 = vigorous effort to escape cold, such as jumping. The sum of the scores recorded within a 60-s period was calculated.

### TRP channel agonist-evoked nocifensive behaviors

DL-Menthol (Sigma-Aldrich) and capsaicin (Nacalai Tesque, Kyoto, Japan) were dissolved in dimethyl sulfoxide (DMSO) as a stock solution (800 mg/ml and 80 mg/ml, respectively). Allyl-isothiocyanate (AITC, Wako), menthol, and capsaicin were diluted in corn oil (Sigma-Aldrich). The mice were allowed to acclimate in a clear acrylic cylinder for at least 40 min after which 20 μl of AITC (0.1%), capsaicin (1.6 μg) or menthol (160 μg) was subcutaneously injected into the plantar surface of the left hindpaw. AITC- and capsaicin-evoked nocifensive behaviors were measured as the durations of consecutive licking and flicking behaviors for 20 min and 5 min, respectively. Menthol-evoked nocifensive-like behaviors (i.e., hindpaw lifting and backwards walking) were scored as for the cold-plate test described above. The sum of the scores recorded within a 5-min observation period was calculated.

### Primary cultures of DRG neurons

Bilateral L1-L6 DRGs were harvested from two freshly killed adult male C57BL/6 J mice. DRGs were incubated for 1 h at 37°C in Hank's balanced salt solution (137 mM NaCl, 5.4 mM KCl, 0.34 mM Na_2_HPO_4_, 0.44 mM KH_2_PO_4_, 5.6 mM D-glucose, 2.4 mM HEPES, 25 mM glucose, pH 7.4) containing 0.3% collagenase and 0.4% dispase. A Percoll (Sigma-Aldrich) gradient was used to separate myelin and nerve debris from DRG neurons as follows: Solutions of 30% and 60% Percoll were prepared with L15 medium. The 30% Percoll was gently layered over the 60% Percoll solution, and the cell suspension over the Percoll gradient. After 10 min of centrifugation at 1800 × g, the cells were harvested from the Percoll interface and suspended in 8 ml L15 medium, then centrifuged again for 5 min at 1800 × g. The supernatant was removed and the cell pellet resuspended in 70 μl Dulbecco's modified Eagle medium (DMEM), containing 10% heat-inactivated fetal bovine serum, penicillin G (100 U/ml), and streptomycin (100 μg/ml), followed by plating onto laminin-coated coverslips (3 mm × 7 mm) and incubation at 37°C. After 4 h incubation, 1.5 ml DMEM was added and the cells were incubated again, this time overnight at 37°C.

### Fluorometric Ca^2+^ imaging

Cultured DRG neurons on coverslips were loaded for 30 min with 5 μM fura-2/AM (Dojindo, Kumamoto, Japan) in Krebs-Ringer solution (140 mM NaCl, 5 mM KCl, 1 mM MgCl_2_, 2 mM CaCl_2_, 10 mM glucose, 10 mM HEPES) containing 0.01% cremophore EL (Sigma-Aldrich). The cells were then washed with Krebs-Ringer solution and transferred to an imaging chamber. The AQUACOSMOS/ORCA-AG imaging system (Hamamatsu Photonics, Shizuoka, Japan) was used to capture the fluorescence images obtained with alternating excitation at 340 and 380 nm and emission at > 510 nm. Emission ratios (F_340_/F_380_) were calculated for each 5-s interval after subtraction of the background. The cells showing the F_340_/F_380_ ratio of over 1.4 at the baseline were excluded. A cellular response to a drug was defined as an increase in the F_340_/F_380_ ratio by more than 0.2 during the 3-min application period. All experiments were performed at room temperature. Oxaliplatin stock solution (5 mM) was prepared in sterile water and further diluted in DMEM to 100 μM before every experiment. AITC (100 mM), menthol (1 M), and capsaicin (100 mM) were prepared as stock solutions in DMSO and diluted in Krebs-Ringer solution.

### Statistical analysis

The data were analyzed using Graphpad Prism and are presented as means ± S.E.M. Statistical significance was calculated by one-way or two-way analyses of variance (ANOVA), followed by the Bonferroni post-hoc test. Time-course data were analyzed by two-way ANOVA for repeated measures, followed by the Bonferroni post-hoc test. In all cases, differences of *p* < 0.05 were considered statistically significant.

## Abbreviations

AITC: allyl-isothiocyanate; ANOVA, analyses of variance; DMEM: Dulbecco's modified Eagle medium; DMSO: dimethyl sulfoxide; DRG: dorsal root ganglia; PKA: protein kinase A; TRP: transient receptor potential; TRPA1: transient receptor potential, subfamily A: member 1; TRPM8: transient receptor potential, subfamily M: member 8; TRPV1: transient receptor potential, subfamily V member 1.

## Competing interests

The authors declare that they have no competing interests.

## Authors’ contributions

All authors read and approved the final manuscript. MZ and KI carried out the experiments and data analysis. HS helped the experiments and data analysis. TN conceptualized the hypothesis, designed, directed the data analysis and provided data interpretation. MZ and TN wrote the manuscript. SK supervised the experiments and finalized the manuscript.
